# Brain Miffed by Macrophage Migration Inhibitory Factor

**DOI:** 10.1155/2012/139573

**Published:** 2012-09-03

**Authors:** Nic E. Savaskan, Günter Fingerle-Rowson, Michael Buchfelder, Ilker Y. Eyüpoglu

**Affiliations:** ^1^Department of Neurosurgery, University of Erlangen-Nuremberg, Schwabachanlage 6, 91054 Erlangen, Germany; ^2^Clinic I for Internal Medicine, University Hospital Cologne, Kerpener Straße 62, 50924 Cologne, Germany

## Abstract

Macrophage migration inhibitory factor (MIF) is a cytokine which also exhibits enzymatic properties like oxidoreductase and tautomerase. MIF plays a pivotal role in innate and acquired immunity as well as in the neuroendocrine axis. Since it is involved in the pathogenesis of acute and chronic inflammation, neoangiogenesis, and cancer, MIF and its signaling components are considered suitable targets for therapeutic intervention in several fields of medicine. In neurodegenerative and neurooncological diseases, MIF is a highly relevant, but still a hardly investigated mediator. MIF operates via intracellular protein-protein interaction as well as in CD74/CXCR2/CXCR4 receptor-mediated pathways to regulate essential cellular systems such as redox balance, HIF-1, and p53-mediated senescence and apoptosis as well as multiple signaling pathways. Acting as an endogenous glucocorticoid antagonist, MIF thus represents a relevant resistance gene in brain tumor therapies. Alongside this dual action, a functional homolog-annotated D-dopachrome tautomerase/MIF-2 has been uncovered utilizing the same cell surface receptor signaling cascade as MIF. Here we review MIF actions with respect to redox regulation in apoptosis and in tumor growth as well as its extracellular function with a focus on its potential role in brain diseases. We consider the possibility of MIF targeting in neurodegenerative processes and brain tumors by novel MIF-neutralizing approaches.

## 1. Introduction

**Figure 1:**
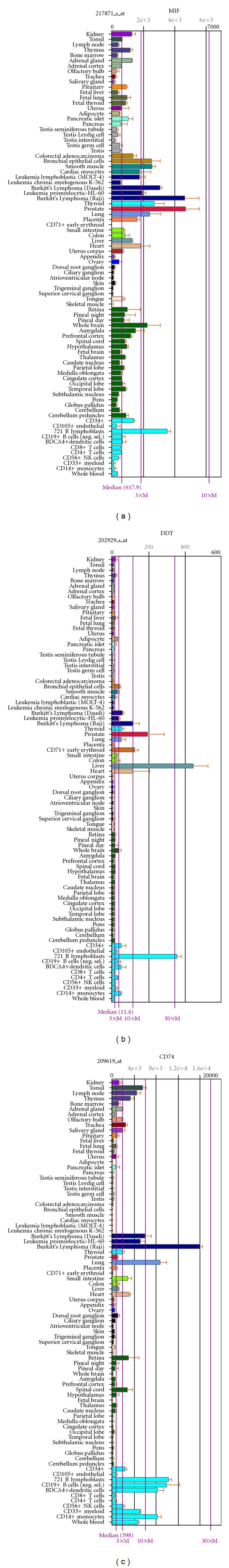
MIF, DDT, and CD74 distribution in human tissues. Comparative analysis of MIF, DDT (MIF-2), and its receptor CD74 expression in various human tissues. For human mRNA expression analysis, the BioGPS database (http://biogps.gnf.org profile graph) with the Affymetrix chip Human U133A was acquired. Note in particular the different expression values of MIF and DDT in brain tissue. For details on the Affymetrix chip analysis, see [37, 38].

Macrophage migration inhibitory factor was one of the first cytokines identified after interferon [1] and represents a key regulator of the immune system (MIF is historically also known as glycosylation-inhibiting factor, GIF) [2, 3]. MIF was initially described as a proinflammatory soluble factor derived from T cells under various conditions such as delayed-type hypersensitivity responses and inflammation guiding site-specific migration of immunocompetent cells [2, 4]. It soon became apparent that MIF possesses immunoregulatory effects and is even constitutively detectable in various body fluids and cells of the mammalian organism. MIF levels are higher at sites of inflammation, within immune and brain cells and various cancer cells (Figure 1). Later, MIF was shown to contribute to neuroendocrine modulation, as a pituitary gland-derived hormone, inflammation, atherosclerosis, cancer development, and cancer progression [5–11]. MIF was first cloned from T cells in 1989, which revealed not only its primary sequence and conserved domains but also led to the discovery that MIF exhibits two catalytic centers, one for thiol-protein oxidoreductase activity and another one for tautomerase activity [12–14]. These findings fueled speculation that MIF was not only a cytokine, but a possible combination of enzyme and cytokine “cytozyme” [12, 13, 15, 16]. Hence, MIF's conserved gene structure and structural homology with D-dopachrome tautomerase (DDT/MIF-2) aroused further speculation surrounding its proposed enzymatic actions and cytokine properties [17, 18]. This enigmatic property of MIF fostered the development of genetic approaches towards a better understanding of its biology in physiology and disease. To date, it is known that MIF induces pleiotropic functions in inflammation, malignant transformation, and endocrine and metabolic processes. In this paper, we focus on MIF-dependent signaling in redox regulation and brain cancer progression and discuss recent findings in MIF neurobiology.

## 2. MIF Structure and Function

The small and highly conserved protein MIF with an approximate molecular weight of 12.5 kDa (human MIF contains 115 aa) does not exhibit any similarities with known cytokines [12, 19, 20]. MIF protein does not require an N-terminal export-specific leader sequence for secretion as it is secreted via an alternative, nonclassical pathway.

**Figure 2:**
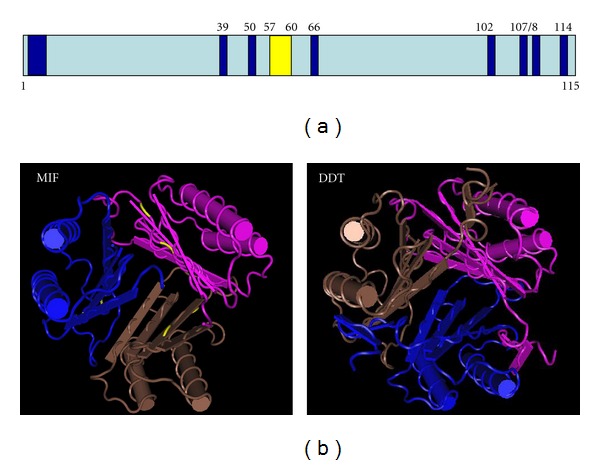
Structural homologies of MIF and DDT. (a) Primary structural scheme of the human MIF gene. Yellow region indicates the CXXC domain, the blue boxed domains indicate the proposed tautomerase/isomerase domains and clustered amino acids (Phe3, Val39, Gly50, Lys66, Asn102, Gly107, Trp108, Phe113, and Ala114) [18]. (b) Structural comparison of human MIF and DDT trimers. The catalytically important CXXC domain is shown in yellow. *β*-sheets are given as arrows, and *α*-sheets are shown as columns. Data were obtained from the NCBI database (http://www.ncbi.nlm.nih.gov/Structure/mmdb/mmdbsrv.cgi?uid=89970) based on the study of [28].

However, MIF contains two conserved domain motifs (Figure 2). The CXXC domain motif (Cys-X-X-Cys at position 56–60) in the center of MIF has been shown to exhibit catalytic activity [21–23]. It is a consensus sequence of proteins of the thiol-protein oxidoreductase superfamily, other members of which include thioredoxins, glutaredoxins, and peroxiredoxins [24, 25]. Common to this enzyme superfamily is that all members are involved in disulfide-mediated redox reactions and glutathione metabolism in which the CXXC domain takes center stage. In the case of MIF, the CXXC domain is potentially involved in forming MIF homodimers and trimers, the most likely active form of MIF [26–28]. Hence, the CXXC domain of MIF has been shown to exhibit low redox catalytic activity *in vitro* (compared to thioredoxin and glutaredoxins) and modulates cellular redox stress responses by elevating the intracellular glutathione (GSH) pool [14, 29–34]. In particular, reactive oxygen species (ROS) induce elevated MIF mRNA and protein expression in neurons, and MIF represents a negative regulator for angiotensin-II-induced chronotropic action and firing in neurons [33, 35, 36]. In addition, MIF has been found to protect from oxidative stress in an ischemia/reperfusion cardiac lesion model [29, 34].

It is worthy to note that the CXXC domain in MIF seems to be essential in facilitating the inhibition of angiotensin II. Evidence for this comes from MIF peptide fragments containing the CXXC domain (ΔMIF^50–65^) which mimic the wild-type MIF action whereas a mutant ΔMIF^50–65^ replacing the second cystine to serine (C60/S60) does not [36]. Redox stress is known to be elevated under conditions of hypoxia or malignant transformation. Hypoxia-inducible MIF elevation has been reported in head and neck cancer cells, pancreas, cervical carcinoma cell lines, and glial tumors [40–43]. Further studies revealed that MIF transcription is induced by hypoxia-inducible factor 1*α* (HIF1*α*) and is physically linked to HIF1*α* through COP9 signalosome subunit 5 (CSN5) interaction [42, 44, 45]. MIF can potentially inhibit apoptosis and p53-mediated growth arrest and its depletion impairs cell proliferation in cancer [6, 7, 46–48]. It was suggested that MIF's action of blocking apoptosis is dependent on its catalytic oxidoreductase activity. However, whether MIF deletion in tumors makes them prone to hypoxia and affects tumor vasculature *in vivo* remains to be thoroughly investigated. First studies already indicate that MIF expression and MIF signalling are associated with tumor angiogenesis [49–51].

The second enzymatic domain of MIF is its enigmatic tautomerase activity which has spurred intensive research on the physiologic substrate and function. In an attempt to identify the enzyme responsible for converting the non-naturally occurring substrate L-dopachrome into dihydroxyindole carboxylic acid (a catalytic step important in biosynthesis of melanin), MIF was purified and subsequently identified by peptide sequencing from bovine lens tissue [13]. Further investigations of the structure of MIF revealed that the tautomerase/isomerase activity is located at the N-terminal portion with a conserved proline residue at position 2 [27, 28, 52–54]. The three-dimensional protein structure of MIF revealed striking similarities with D-dopachrome tautomerase (DDT/PPT2) although MIF shares solely ~30% amino acid sequence homology with DDT [28, 53] (Figure 2). These findings led to various enzymatic and mutational investigations identifying the N-terminal portion of MIF as essential for tautomerase activity [15, 16, 55]. However, since the finding of MIF's *in vitro* tautomerase activity investigations have focused on the identification of its physiological substrate and biological role which is still ongoing. Genetic studies in the meantime revealed that catalytically dead mutants still exert MIF-specific functions. Moreover, tautomerase-null MIF knock-in mice compensate the MIF gene deletion (MIF^−/−^ or MIF null mutant) phenotype which leads to the argument that the tautomerase activity may be possibly dispensable *in vivo* [18, 56, 57]. MIF's highly conserved substrate pocket may represent a vestigial relict reflecting its ancestral origin in innate immunity and be dispensable at least for its function in promoting cellular growth and tumorigenesis *in vivo* [11, 47, 58, 59]. However, the catalytically dead MIF mutant (P1G-MIF) shows reduced binding to some protein interaction partners, such as its cell surface receptor CD74 and the c-jun amino-terminal kinase activator Jab1/CSN5. This indicates that the N-terminal proline and the catalytic pocket may play a role in protein-protein interaction of MIF with its binding partners [57]. Noteworthy were findings reporting more pronounced phenotype and defects in CD74 knock-out mice (MIF receptor) than in solely MIF-deficient mice [59–64]. This led to the hypothesis that more MIF-like ligands acting on CD74 receptor may exist. The group of Bucala and colleagues recently identified D-dopachrome tautomerase (DDT) as a MIF-like cytokine with overlapping functions [65]. Neutralizing antibodies against DDT can protect mice from lethal endotoxic shock to a comparable extent as MIF neutralization, by reducing circulating TNF-*α*, IFN-*γ*, IL12, and IL-1*β* [5, 60, 65–68]. It has subsequently been suggested to redefine DDT as MIF-2 due to their structural homologies and functional resemblance with data on DDT knock-out mice and combined neutralization studies to unravel this renaming.

## 3. MIF Distribution in the Brain

**Figure 3:**
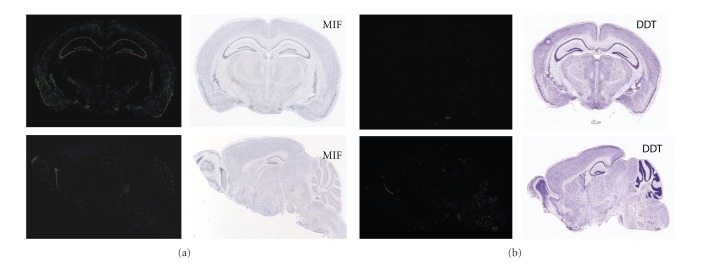
MIF and DDT distribution in the brain. Representative in situ hybridization images of MIF mRNA (a) and DDT mRNA distribution (b) in adult mouse brain (left) with consecutive counterstained brain section (Nissl stain, right). Upper panels of (a) and (b) represent coronal plane; lower panels show sagittal plane. Data were provided from the Allan Brain Atlas website (http://www.brain-map.org/), and the Brain Explorer 1.3 software was utilized for the visualization of gene expression [39].

Distribution and microarray expression profiles (BioGPS analysis) of MIF, DDT, and their joint receptor CD74 already suggest spatial overlapping as well as ancillary functions (Figure 1). MIF is widely expressed in the body and shows high levels in lymphocytes, thyroid, prostate, placenta, and lungs. In the murine brain, MIF transcripts and protein are mainly present in the cortex, hippocampus, and pituitary gland [5, 69] and thus differ in distribution and expression level in comparison to DDT (Figure 3). In particular, MIF immunoreactivity has been found in neurons of the hippocampus within fiber structures and terminals such as the mossy fibers of the dentate gyrus and in dendrites of the hippocampal CA regions [69]. Furthermore, MIF is upregulated in neurons and in macrophages following intracranial LPS stimulation. Interestingly, MIF is also found in microglial cells, the resident macrophages of the brain as well as in cerebrospinal fluid (CSF), and shows elevation after experimental LPS treatment, too. Moreover, MIF pretreatment can reduce the number of invading microglial cells and macrophage into allogeneic fetal mesencephalic grafts in rodents [70]. However, this MIF treatment did not affect the outcome on graft function and survival leaving the potential of MIF as a neuroimmune modulator in Parkinson's disease open. It has recently been shown that MIF can promote the growth of neural progenitor cells *in vitro* [71], indicating already a growth-promoting effect in particular cell populations. Contrary to such growth promoting effect is one report on elevated MIF levels in the cerebrospinal fluid of Alzheimer patients and the beneficial effects of MIF inhibition after amyloid *β* protein-induced neurotoxicity *in vitro* [72]. As indicated above, MIF may function in a context-dependent manner with various effects on different neural and glial cells. The presence of MIF in hippocampal structures which are prone to glucocorticoid-induced tissue damage has led to speculations of MIF and its association with glucocorticoid action under normal and pathophysiological processes.

## 4. MIF Signaling, Glucocorticoids, and Metabolism

MIF was one of the first cytokine-mediated activities derived from T cells described. It then became apparent that MIF is also expressed by monocytes/macrophages and signals in both an autocrine and paracrine manner [2, 4]. Gene-targeting experiments and neutralization approaches affirmed its upstream role in the inflammatory cascade promoting proinflammatory mediators such as TNF-*α*, IL-12, IL-1*β*, and PGE_2_ [7, 59, 60]. MIF's role as an autocrine innate immune regulator has been exemplified by its “auto-loop” route through TNF-*α*, which in turn leads to further MIF secretion in macrophages [73]. Thus, it became apparent that MIF follows two signaling principles. First, MIF executes its biological function as a secreted molecule requiring specific receptor(s) at the cell surface of its target cells, that is, transcellular signaling. Secondly, MIF acts as an intracellular or autocrine signaling molecule with catalytic activity and specific binding partners due to its structural features (intracellular domains and mechanisms; see section above).

**Figure 4:**
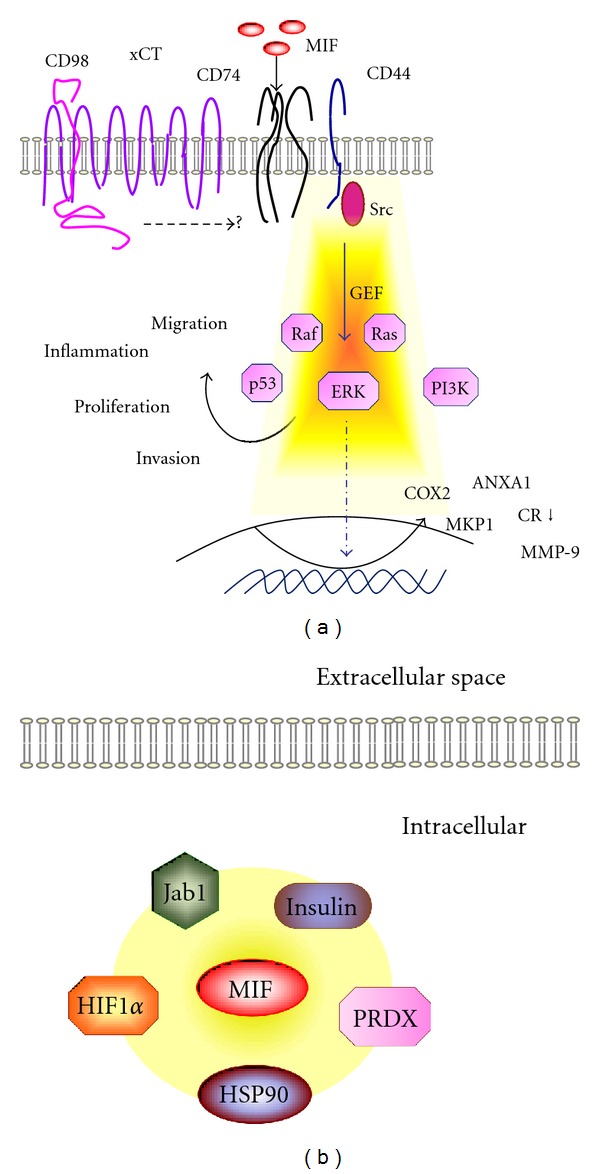
MIF receptor signalling and downstream effectors. (a) Schematic model of receptor-mediated MIF signalling involving CD74 and CXCRs. The involvement of the glutamate antiporter xCT (system x_c_^−^, xCT forms a heterodimer with CD98 as indicated) in CD74/CD44-dependent signalling is proposed, indicated by the dotted arrow. (b) MIF binding partners with link to brain cancer. Note that the indicated MIF-binding partners given in the scheme are far from complete. Abbreviations used: COX2, cyclooxygenase 2; ERK, extracellular signal-regulated kinases; GC, glucocorticoids; GR, glucocorticoid receptor; GEF, guanosine exchange factor; HIF1*α*, hypoxy Jab1, Jun-activation domain-binding protein-1; MKP1, mitogen-activated protein kinase phosphatases; MMP-9, matrix metallopeptidase or type IV collagenase/gelatinase B; PRDX, peroxiredoxin; Src, sarcoma protooncogene.

The identification of MIF's receptor-mediated signaling gave rise to a hub for the discovery of intracellular and extracellular interaction partners and functions [63, 74–79]. To date receptor-mediated MIF signaling has been identified through the cell surface receptor complexes CD74 (CD74/invariant chain—CD44 signaling complex), CXCR2, CXCR4, and CD74-CXCR2/4 [63, 74, 76, 80] (Figure 4). Especially the structural homology of the canonical CXCL8 ligand, a so called pseudo-(E)LR motif present in MIF and binding to CXCR2 and CXCR4 qualified MIF as a non-cognate chemokine ligand [27, 63, 81]. These receptors bind MIF to the surface of cells and mediate activation of extracellular-regulated mitogen-activated protein (ERK-MAP), phosphatidylinositol 3/protein kinase B (PI3K/AKT), and Src-tyrosine kinases through CD44, already indicating the presence of a link to oncogenic signaling utilized by cancer cells (Figure 4).

In particular, MIF impacts macrophage and lymphocyte functions and thereby regulates innate and acquired immunity [73, 82, 83]. In mice, MIF was cloned as an immunoregulatory peptide from the pituitary gland and was shown to specifically counteract glucocorticoid effects such as suppression of TNF-*α*, IL-8, and IL-1*β* secretion [5, 84, 85]. Moreover, MIF's impact on the innate immune system can be fatal in lethal endotoxic shock by counteracting the protective effects of glucocorticoids at various levels [86, 87]. Glucocorticoids and steroid analogues such as dexamethasone are widely used and are most effective anti-inflammatory drugs, acting through various mechanisms and recruiting downstream effectors such as NF*κ*B, histone deacetylase 2 (HDAC2), *α*1*β*1 integrin, and phospholipase A2 (PLA2) [88, 89]. In particular, glucocorticoids have been used for decades for the treatment of various neuroinflammatory, neurotrauma, and neurooncological disease conditions. One reason lies in that glucocorticoids are one of the most powerful classes of agents in reducing tumor-associated edema and tissue swelling and can thus reduce the incidence of fatal herniation in space occupying lesions to a certain extent. MIF in this pathway is therefore of clinical significance.

MIF counteracts glucocorticoid signalling by decreasing I*κ*B levels leading to NF*κ*B activation, upregulates PLA2, and downregulates MAP kinase phosphatase 1 [86, 87, 90]. The bell-shaped MIF regulation by glucocorticoids is worthy of note with increased MIF release from monocytes/macrophages at low physiological amounts of glucocorticoids and inhibited MIF release at high glucocorticoid concentrations [84, 91]. In this manner, MIF inhibition offers an alternative strategy for anti-inflammatory therapy in neuroinflammation such as multiple sclerosis and Guillain-Barré syndrome, although the effects of MIF on prescribed glucocorticoid analogues in patients require further consideration. Hence, MIF can control glucose catabolism in muscle cells by elevating the level of the key enzyme phosphofructo-2-kinase leading to lactate production [92]. MIF also modulates downstream AMP-activated protein kinase effects in cardiac cells such as the glucose transport function [93]. Whether MIF upregulates phosphofructo-2-kinase and glycolysis in brain tumor cells with subsequently increased lactate release has not yet been tested. Since the Warburg effect is one characteristic feature of malignant gliomas (i.e., primary brain tumors derived from glial and precursor cells), further investigation into the metabolic effects of MIF in brain tumor cells would be highly desirable.

## 5. MIF Links Inflammation with Cell Cycle Regulation

MIF has a central role as monocytes/macrophages in the global regulator of monocyte/macrophage-derived cytokines. It is an interesting finding that distinct thresholds of MIF affect monocytes/macrophages differentially. At low concentrations MIF induces the release of TNF-*α*, IL-12, IL-1*β*, and PGE_2_ and, in a distinct difference from other “common” cytokines, involves MAPK, Akt, and PI3K activation and regulation of Jab1 and p53 [6, 7, 59, 94, 95]. In particular, the latter is involved in the resolution of inflammation by inducing p53-dependent, activation-induced cell death [6]. High and sustained MIF action, for instance, in chronic inflammation, also promotes the release of macrophage effector cytokines such as TNF-*α*, IL-12, IL-1*β*, and PGE_2_. On the other hand it also prevents cytoplasmatic accumulation of the tumor suppressor gene p53, thus inhibiting apoptosis (Figure 4). This peculiarity of MIF caught the attention of the cancer research field. Bypassing p53-mediated growth arrest is an important feature of cancer cells and of a tumor promoting microenvironment. TP53, the human gene encoding the p53 protein, mutates at a high frequency (approx. 30%) in adult malignant gliomas and glioblastomas. The increased expression of MIF in malignant gliomas is of particular interest since MIF suppresses p53-dependent signaling and thereby enhances susceptibility to further oncogenic mutations. Hence, MIF interacts with Jab1/CSN5 and negatively regulates the cullin-1-containing ubiquitin E3 ligase complex with effects on p27- and E2F1-3-dependent cell cycle control [96, 97]. Conversely, loss of MIF in a p53-deficient background leads to uncoupled DNA damage checkpoint response, thereby aggravating tumorigenesis in p53^−/−/MIF−/−^ mice [96]. It has recently been shown that the chaperone HSP90 stabilizes MIF for E3-ubiquitin-ligase-dependent proteasome degradation in various tumor cells, leading to increased MIF levels even under siRNA-mediated transcriptional silencing [98]. This regulatory protein stabilization feature secures persistent MIF action in cancer cells independent of transcriptional and translational levels.

## 6. MIF, Brain Tumors, Angiogenesis, and Tumor Microenvironment

MIF is produced by neuroendocrine and immune tissues and possesses several features that allow it to be classified as a neuroendocrine mediator [5, 99]. This cytokine has glucocorticoid-antagonist properties within the immune system and participates in the regulation of several endocrine circuits under physiological conditions. Further, initial *in vitro* studies indicate a growth-promoting activity of MIF on neural progenitor cells [71]. In this context, MIF controls the site-specific migration of the immunocompetent cells of the brain, the microglia. These cell entities are considered to be the resident macrophages of the brain and are involved in almost all pathophysiological mechanisms, including trauma, autoimmune and neuroinflammatory disease, and brain tumors. The precise role of these immunocompetent cells of the CNS in tumor progression is subject of much controversy since its specific role is not yet completely understood. Immunological “escape mechanisms” could play a decisive role in tumor invasion and proliferation.

**Figure 5:**
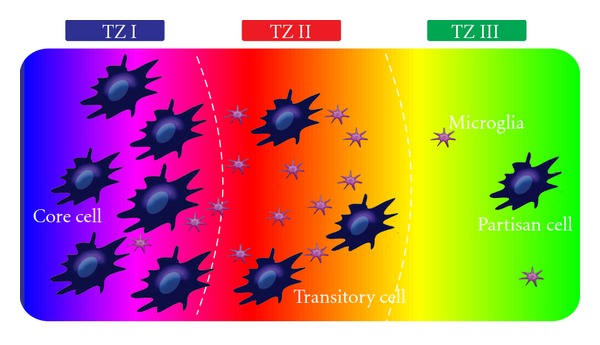
The brain tumor microenvironment, heterogeneous tumor zones and MIF actions. Conceptual framework depicting the metabolic and immune cell complexity of malignant brain tumors, (glioblastomas, GBM) is given as a simplified model classifying the tumor into three distinct tumor zones (TZ1–TZ3). Tumor Zone 1 (TZ1) consists of the main tumor—bulk and core glioma cells, corresponding to contrast enhancing regions in MRI images. MIF is mainly produced in TZ1 and secreted into the extracellular space. TZ2 represents the area of perifocal edema, which is characterized by its specific proangiogenic microenvironment and presence of transitory glioma cells. In addition, this tumor zone shows pronounced accumulation of microglial cells, which also infiltrate TZ1. TZ3 is the most awkward zone for therapeutic intervention, since this tumor zone consists mainly of healthy brain parenchyma. However, isolated glioma-initiating cells termed partisan cells colonize TZ3 and are most probably responsible for tumor recurrence following surgery. TZ2 is probably biologically most active, influencing TZ1 and TZ3 by tumor-derived metabolites impacting the immune system, angiogenesis, and cell fate.

The association of MIF with the progression of malignant brain tumors places this cytokine in center stage [100, 101]. It is suggested that brain tumors secrete MIF to control the activity of accumulating tumor-promoting cells, which in turn might have inductive tumor-progressive as well as proangiogenic effects [58, 101]. Thus, based on its localization and functional features, MIF would be well in a position to execute important control of the tumor microenvironment. A conceptual framework has been sketched to reflect the metabolic and immune cell complexity of brain tumors in a simplified model classifying the tumor into three distinct zones (Figure 5). Although each border may depict a smooth shift into the next transition zone, Tumor Zone 1 (TZ1) consists of the main tumor—bulk, corresponding to contrast enhancing regions in clinical MRI settings. Here, MIF is mainly produced and secreted into the surrounding tissue. TZ2 represents the area of perifocal edema, which is characterized by its specific proangiogenic microenvironment and transitory glioma cells. Apart from these cells, there is a pronounced accumulation of microglial cells, which also infiltrate the TZ1. The TZ3 is the most challenging and intractable zone for therapeutic intervention, since this zone consists mainly of healthy brain parenchyma. However, isolated glioma-initiating cells termed partisan cells colonize TZ3 and are most probably responsible for tumor recurrence following surgery. The TZ2 is probably biologically most active, influencing TZ1 and TZ3 through tumor-derived metabolites impacting the immune system, angiogenesis, and cell fate. With regard to MIF, however, production and secretion of MIF occur in TZ1, while its receptors are mainly expressed by microglial cells in TZ2 and on glioma cells themselves. MIF could therefore act in a dual fashion both as an autocrine factor as well as a tumor-derived factor which influences the immune micromilieu (Figure 5). Another relevant aspect is that malignant gliomas secrete neurotoxic concentrations of the oncometabolite glutamate as a consequence of their metabolic alterations, and increased glutathione needs [102, 103]. Further, glutamate stimulates the migration and activation of microglial cells [104]. This aspect has not been given much attention from a neurooncological point of view. The metabolic cytokine crosstalk reveals its clinical implication: CD44 as coreceptor of CD74 is also a regulatory component of the glutamate transporter xCT controlling cancer redox state [105]. Additionally, as a specific surface cell receptor in mesenchymal stem cells, CD44 regulates the vascular architecture of highly vascularized tumors such as malignant gliomas through the activation of these stem cells, thereby playing a possible role in their progression. Nevertheless, it needs to be unambiguously demonstrated whether MIF is primarily effective in an autocrine or intracellular manner in malignant gliomas. Thus, further studies on this matter will be decisive for future MIF-neutralizing approaches. Two approaches are available in experimental and clinical studies for the therapeutic targeting of MIF. Firstly, MIF-neutralizing antibodies have been experimentally tested in a murine arthritis model and in rodent glomerulonephritis models with promising efficacy [106–108]. Along the same line, CD74-neutralizing antibodies have been applied to B-cell malignancies, although comparable data of these approaches are missing. Additionally, soluble CD74 molecules have been isolated *in vitro*. Secondly, there are now effective, small-molecule MIF antagonists available, with ISO-1 being the most widely accepted one [78, 108]. Based on these findings further small compound library screenings and computational drug design studies are now underway. This approach will probably identify promising small-molecule MIF inhibitors in the future. Due to the lack of immunological responses, low-molecular-weight inhibitors are so far most promising for MIF-neutralizing approaches in humans.

Considering data from clinical studies as well, MIF expression also has predictive values, as patients with malignant gliomas and high MIF expression levels show worse prognosis and earlier tumor recurrence [109]. Interestingly, MIF abundance is associated with increased microvessels and elevated IL-8 expression. Moreover, the MIF receptor CD74 has been shown to contribute to temozolomide resistance [109, 110]. Taking all these facts into account, the underlying molecular mediators and metabolites and immunological crosstalk remain only partially understood despite the central role of dysregulated metabolism in brain tumors. A comprehensive understanding of the dynamics and hierarchy of MIF as a glioma-derived oncometabolite as well as immunological and vascular consequences is therefore critical in identifying effective drug targets in the development of multimodal managements of brain tumors. In order to achieve this target, a detailed analysis of MIF action in this disease with high unmet medical need appears mandatory. Future studies will show whether available MIF and CD74 receptor inhibitors could be efficiently used in our armamentarium against malignant brain tumors.
